# Assessment of vape shop built environment: airborne nicotine, particulate matter, ventilation, hazard identification, workplace practices, and safety perceptions

**DOI:** 10.1093/annweh/wxaf018

**Published:** 2025-06-19

**Authors:** Toluwanimi M Oni, Balaji Sadhasivam, Evan L Floyd

**Affiliations:** Department of Occupational and Environmental Health, Hudson College of Public Health, University of Oklahoma Health Sciences, Oklahoma City, OK 73104, USA; Department of Occupational and Environmental Health, Hudson College of Public Health, University of Oklahoma Health Sciences, Oklahoma City, OK 73104, USA; Department of Occupational and Environmental Health, Hudson College of Public Health, University of Oklahoma Health Sciences, Oklahoma City, OK 73104, USA

**Keywords:** electronic cigarettes, exposure assessment, nicotine, particulate matter, safety, vape shops

## Abstract

Vape shops are established to sell electronic cigarette (EC) devices, e-liquids or e-juices, and other related accessories. EC use is prominent in vape shops and indoor EC use has been associated with elevated levels of nicotine and particulate matter (PM). This study assessed health and safety conditions, practices, building characteristics, nicotine, and PM concentrations in vape shops during business hours. Sixty-four vape shops were visited but only 15 vape shops consented to participate in this study. The majority of the vape shops had general ventilation (100%) and lounge areas (60%). No workers were observed not to use any personal protective equipment (PPE) such as gloves, aprons, face masks, etc. The mean and standard deviation of the shop volume, air flowrate, and air exchange rate were 12.361 ± 12.990 ft^3^, 1.203 ± 1.584 ft^3^/min, and 5.8 ± 2.8 h^−1^, respectively. The mean and standard deviation of the time-averaged concentration of nicotine, PM_2.5_, respirable PM, and total PM were 3.92 ± 3.73, 32.01 ± 25.85, 36.03 ± 30.91, and 43.67 ± 34.78 ug/m^3^, respectively. The nicotine, PM_2.5_, respirable PM, and total PM levels were significantly below their respective occupational or ambient guideline limits (*P* < 0.05). The vape shop environments in this study did not appear to pose a significant risk of second-hand exposure to elevated levels of airborne nicotine and PM during business hours.

**What’s Important About This Paper?**

Few studies have assessed the vape shop built environment and vape shop workers’ health and safety. This study found nicotine and particulate concentrations to be below occupational exposure limits. Workers recognized nicotine and e-liquids were hazardous, but did not identify other hazards or factors responsible for hazards present in their workplace. Vape shops have a variety of hazards that need to be addressed to protect worker health and safety.

## Introduction

Electronic cigarette (EC) use is widespread and because of their popularity, many retail outlets have emerged to provide ECs, EC-related products and services to their numerous customers (Callahan-Lyon 2014; Cheng 2014; Lee et al. 2018). Initially, ECs and accessories sales mostly occurred over the internet (Lee and Kim 2015), but now they are sold in retail “brick-and-mortar” stores which are called vape shops (Sussman et al. 2014; Lee et al. 2018; Berg et al. 2020; Attfield et al. 2022). Many vape shops have lounges with television sets, couches, and organize vape cloud-making competitions to encourage customer interactions and sales (Sussman et al. 2016).

The number of vape shops in the United States has increased rapidly. In 2013, an estimated 3,500 vape shops were in operation (Klein 2013). In 2014, the number of vape shops grew to approximately 5,000 (Richtel 2014) and by 2015, rose to 9,945 (Dai and Hao 2017). Oklahoma is a unique location for studying vape shops because the per capita rate has been approximately 5 times that of the general US population. This was derived from Richtel et al. who reported 300 vape shops in Oklahoma (6%) out of a total of 5,000 in the United States (Richtel 2014). In 2014, Oklahoma’s population was approximately 1.2% of the US population but housed 6% of all vape shops.

EC aerosol is a mixture of liquid droplet particulate matter, vapor from the droplets, and trace levels of pyrolysis products from vaporization (Pratte et al. 2016; Marcham and Springston 2019). The primary components of EC aerosol are similar to the primary components in the source solution, which are propylene glycol, glycerol, nicotine, and flavorings (Margham et al. 2016; Pratte et al. 2016; Li et al. 2021b). The particulate matter in the EC aerosol is mostly comprised of liquid droplets that readily evaporate when the environment is not saturated with contaminant vapor (Martuzevicius et al. 2019). Since EC use is common within vape shops, if not encouraged, (Nguyen et al. 2019; Zhang et al. 2020), elevated levels of propylene glycol, glycerol, nicotine, and flavorings is anticipated in the air within vape shops. Indeed, elevated nicotine and PM levels have been reported within vape shops (Chen et al. 2018; Nguyen et al. 2019; Li et al. 2021a). Other studies have reported higher nicotine and PM levels in vape shops (Floyd 2017; Son et al. 2020; Li et al. 2021a). This shows the potential for contaminants from vape shops to infiltrate into neighboring shops.

The study by Floyd (2017) was conducted in Oklahoma and investigated infiltration potential from vape shops to neighboring shops. They observed alarmingly high PM levels during relatively brief visits to vape shops and elevated surface nicotine levels. They observed that PM in non-adjacent reference shops were significantly lower than in vape shops, but adjacent shops were not significantly different from the vape shops. It is noteworthy that they did not measure airborne nicotine in any of the study locations (Floyd 2017). Although most studies have found that PM and nicotine concentrations were well below their respective Occupational Safety and Health Administration (OSHA) occupational exposure limits (OELs), the Floyd (2017) study performed in Oklahoma City suggested full shift exposures were likely to far exceed OELs for respirable PM and possibly nicotine. This is a concern because exposure to chemicals and PM is not usually a reasonably anticipated exposure for retail outlets and is likely an occupational hazard that is going unrecognized and uncontrolled in these workplaces. Thus, vape shop workers (VSWs) and customers may be exposed to elevated nicotine and PM concentrations in vape shops more than in other retail environments.

VSWs may also experience spillage of e-liquids containing nicotine (Garcia et al. 2016). Some VSWs may not have access to or use personal protective equipment (PPE) and safety equipment (Garcia et al. 2016; Attfield et al. 2022). VSWs also tend not to use general ventilation within the shops even when available (Nguyen et al. 2019; Son et al. 2020; Attfield et al. 2022), though such ventilation may help reduce indoor pollutant concentrations. These hazards may result in adverse health outcomes among the VSWs.

Environmental nicotine and PM concentrations in vape shops have only been assessed in a few studies (Son et al. 2020; Li et al. 2021a; Attfield et al. 2022) and these studies had small sample sizes (<10 vape shops each). Furthermore, very little is known about the indoor environmental conditions (Nguyen et al. 2019), and health and safety practices in vape shops. Since the number of vape shop in Oklahoma is ~5× greater per capita than the rest of the United States and prior work by this research group (Floyd 2017) found alarmingly high levels of PM in Oklahoma City vape shops, this study sought to characterize full day exposures of VSWs to PM and airborne nicotine within the Oklahoma City area.

Therefore, this study sought to be the first to assess nicotine, PM, and occupational hazards in vape shops after the COVID-19 pandemic was declared to be over in May 2023 by both the World Health Organization (WHO) and the US Department of Health and Human Services.

## Methods

A total of 71 vape shops in the Oklahoma City Metropolitan Area were identified through a Google search using keywords “vape shops” and “vapor shops.” Only shops that met the following criteria were considered for inclusion in this study: exclusive sale of vapes and vape products, no sale of traditional tobacco products, not physically connected to other vape shops, and not physically connected to a tobacco retailer. The criteria were selected to avoid potential cross-contamination of indoor air in the study locations with nicotine from other vape shops and tobacco smoke. Of the 71 identified vape shops, 64 met the inclusion criteria. Of the 64 eligible shops, 15 vape shops and 16 VSWs agreed to participate in the study. Each eligible shop was approached in person to obtain written consent to participate in this study. For environmental measures, consent was obtained from the manager or owner. For survey-based data, written informed consent was obtained from the VSWs. The number of VSW participants was limited to 1 per vape shop. One vape shop did not give consent for environmental measures but the VSW chose to participate. This study was approved by the Institutional Review Board (13399) from the University of Oklahoma Health Sciences, Oklahoma City, Oklahoma. All vape shops assessed in this study were visited between May and September 2023. Questionnaires were interviewer-administered to participants to gather information on sociodemographic characteristics, health and safety practices, occupational history, practices, conditions, and EC use behavior.

Onsite observation of the vape shops was performed with the aid of a checklist that identified the presence of: visible PM or haze (eg visual perception or activities visibility emitting aerosol like vaping), odors and flavors (eg perceived by the researcher compared against odors outside the shop), lounge areas, air filtration devices, ceiling fans, other ventilation devices, perceptible sources of air pollution within the vape shops, good hygiene conditions, personal protective equipment (PPE), first aid kits, and fire extinguishers. Air flow rates at the air vents were measured using a balometer (TSI ALNOR Air Flow Capture Hood; model EBT731), and shop volume was used to calculate the air exchange rate (h^−1^).

Onsite observations were performed at the beginning of business day and verified at close of business.

Air samples were collected over the duration of each shop’s business hours. Sodium bisulfate-treated glass fiber filters (Whatman glass microfiber filters, Grade GF/A, 47 mm, Cytiva) were coupled with GRIMM portable aerosol spectrometers (Grimm Aerosol Technik Ainring GmbH & Co. KG, Germany) to measure PM levels in real-time and collect nicotine on the treated filter. The GRIMM portable aerosol spectrometers were placed in areas where interactions between VSWs and customers were most frequent and remained in a single location throughout the sampling period. Five (5) field blank samples were also collected. The sodium bisulfate-treated filters were analyzed for nicotine content using an adapted and simplified gas chromatography (GC) described in another study (Oni et al. 2024). The lower detection limit for the GC was 0.13 µg/mL.

Non-detect values were assigned half the value of the lower detection limit of the GC technique for the quantification of nicotine (EPA 1991). Summary statistics were tabulated for onsite observations, and nicotine and PM measurements with the geometric mean (GM) representing the measure of central tendency. The 95th percentile values of the nicotine and PM measurements were compared to OSHA and ACGIH OELs to determine compliance. In addition, the 98th percentile of the PM values was compared to the US EPA 24-h PM_2.5_ standard of 35 µg/m^3^. The Shapiro–Wilks test was used to determine the distribution of airborne contaminant concentrations. Spearman’s correlation was performed to assess the correlation between nicotine and total PM concentrations. Stepwise multiple regression analyses were also performed to determine the predictors of nicotine and total PM concentrations in the vape shops.

## Results

The majority of VSWs in this study (*n* = 16) identified as male, racially white, less than 29 years old (Supplementary Fig. S1), completed only high school, and earned between $10,000 and $29,999 annually. Mean ± SD age of VSWs in this study was 32.3 ± 10.3 years and ranged between 23 and 56 years. Most VSWs reportedly worked either less than 8 h (38%) or more than 8 h (38%) while 25% worked for 8 h per day (Supplementary Fig. S2). Further characteristics of participants are presented in Supplementary Tables S1 and S2, respectively.

The VSWs in this study had reportedly vaped for an average of 6.75 ± 5.35 years with a range of 0 to 22 years. The number of EC devices currently used by the VSWs ranged from 0 to 20 with an average of 3.5 ± 5.3. The average concentration of nicotine vaped by the VSWs was 30.7 ± 23.1 mg/mL and ranged between 3 and 50 mg/mL. The majority of VSWs (88%) were EC users and used their EC devices at work but 13% were non-EC users. ECs (88%), cigarettes (13%), and cigars (6%) were the popular source of nicotine regularly used by VSWs. When asked about their smoker status, 75%, 13%, and 13% of VSWs were former smokers, current smokers, and never smokers, respectively (Supplementary Fig. S3). Further details about EC use by VSWs are presented in Supplementary Table S3.

Workplace practices and conditions among VSWs are presented in Table 1. The majority of the VSWs (94%) reported that customers were permitted to vape within their workplace. Most VSWs (88%) were allowed to vape at work. None of the VSWs used any respiratory PPE. When asked if there were activities that negatively affected the indoor air quality in their workplaces, 63% of VSWs replied “No.” Of the 38% that responded “Yes,” vaping was the most reported air pollution activity by the VSWs (25%) followed by air pollution from adjacent shops (13%) and dabbing *tetrahydrocannabinol* or cannabis in store (6%) (interviewees were allowed to provide more than one response). Furthermore, 75% of VSWs felt that their workplace environment did not have a negative impact on their health and well-being. When asked to rate the indoor air quality in their workplaces from 1 to 10 (1 = poor and 10 = excellent), the average VSW’s indoor air-quality rating was 8 ± 1, ranging from a score of 6 to 10.

**Table 1.** Workplace practices and conditions among vape shop workers.

Workplace practices and conditions	No (%)	Yes (%)	Not sure (%)
Are customers allowed to vape within this workplace?	6	94	–
Are employees allowed to vape within this workplace?	13	88	–
Do you use any respiratory Personal Protective Equipment (PPE) within this workplace?	100	–	–
Are there activities that negatively affect the indoor air quality in this workplace?	63	38	–
Do you feel your workplace environment has a negative impact on your health and well-being?	75	19	6

Commonly reported hazards, factors causing hazards, and hazard control measures in the VSWs’ workplaces are shown in Table 2. The majority of VSWs (63%) stated that there were no hazards in their workplaces but some VSWs reported electrical hazards, chemical handling, and slips, trips, and falls to be present. Most VSWs (63%) could not identify factors responsible for hazards in their workplaces, but some identified: customers modifying EC devices, simply performing regular work duties and carelessness. Half of the VSWs (50%) reported that they did not have any hazard control measures in their workplaces. Hazard mitigation strategies that were identified included: maintaining good hygiene, using PPE, following safety procedures such as restricting customers from accessing areas behind the counters, asking questions before repairing customers’ devices, using proper tools, and paying attention to surroundings. Furthermore, when asked about who instituted hazards prevention and control measures in their workplaces, the most common response (31%) reported “Self-and/or co-workers,” 19% stated that their “employers alone,” and 13% reported a combination of “employer/self and/or co-workers” were responsible.

**Table 2.** Commonly reported hazards, factors, and hazard control measures in vape shop workers’ workplaces.

Common hazards, factors, and hazard control measures		Proportion of respondents (%)
Common hazards in your workplace	Electrical hazards	19
	Chemical handling	13
	Slips, trips, and falls	13
	None	63
Factors mostly responsible for hazards in your workplace	Customers modifying devices	13
	Performing regular duties	13
	Carelessness	6
	None	63
Hazard control measures	Maintaining good hygiene (Handwashing, cleaning workstations)	31
	Using PPE	25
	Following safety procedures	13
	Asking questions before repairing customers’ devices	6
	Using work-related tools	6
	Paying attention to surroundings	6
	None	50
Who puts in place hazard prevention and control methods?	Employer	19
	Employer/Self and/or co-workers	13
	Self and/or co-workers	31
	None	38

VSWs’ knowledge and perception of workplace health and safety are presented in Table 3. The majority of VSWs (63%) stated that elevated levels of nicotine in the workplace were a hazard. Elevated PM levels in the workplace were also considered to be hazardous by most VSWs (88%). Opinions were split regarding the hazardous nature of handling bottles or containers of nicotine or e-liquid. However, 63% of VSWs believed that having direct contact with nicotine solutions and e-liquids while working could be hazardous.

**Table 3.** Knowledge and perception of workplace health and safety among vape shop workers.

Health and safety knowledge	No (%)	Yes (%)
Can elevated levels of nicotine in your workplace be considered as a hazard?	38	63
Can elevated levels of PM in your workplace be considered as a hazard?	13	88
Can handling bottles or containers of nicotine or e-liquid be considered as a hazard?	50	50
Can having direct contact with nicotine solutions and e-liquids while working be considered as a hazard?	38	63

Observed indoor characteristics of the vape shops are presented in Table 4. There was no visible PM or haze in any of the vape shops and odors or flavors were not sensed in a majority (80%) of the vape shops. Vaping was observed in 60% of the shops at the time of visit. All vape shops had general ventilation. However, air filtration devices, ceiling fans, and other ventilation types (oscillating fan and floor blower) were also present at 7%, 13%, and 13% of vape shops, respectively. Most (60%) of the vape shops had a lounge area for customers to hang around and of these vape shops with lounges, among which most had open lounge areas. PPE, including face masks, aprons, gloves, and goggles, were not sighted in any of the vape shops. First aid kits and fire extinguishers were each present in only 7% of vape shops.

**Table 4.** Indoor characteristics of vape shops.

Characteristics	Yes (%)	No (%)
Presence of visible PM or haze	0	100
Presence of odors or flavors	20	80
Presence of lounge area	60	40
Partially or fully enclosed lounge area	22	78
Presence of air filtration devices	7	93
Presence of ceiling fans	13	87
Presence of other ventilation devices	13	87
Perceptible sources of air pollution	60*	40
Good hygiene condition	93	7
Presence and use of PPE	0	100
Presence of first aid kits	7	93
Presence of fire extinguishers	7	93

^*^ Vaping was the only source of air pollution observed during onsite observation.

Shop volume, air flow rate, and air exchange rate (AER) for the vape shops are shown in Supplementary Table S4. The shop volume ranged from 98 to 1,590 m^3^, with a median size of approximately 250 m^3^ and mean of 350 m^3^. The mean ± SD (median) for air flow rate (m^3^/min) was 34.1 ± 44.9 (26.3) and ranged between 0.40 and 178 m^3^/min. The mean ± SD for AER was 5.8 ± 2.8 h^−1^ with a median of 6.1 h^−1^ and range of 0.1 to 9.7 h^−1^.

## Nicotine and PM concentrations

**Fig. 1.**
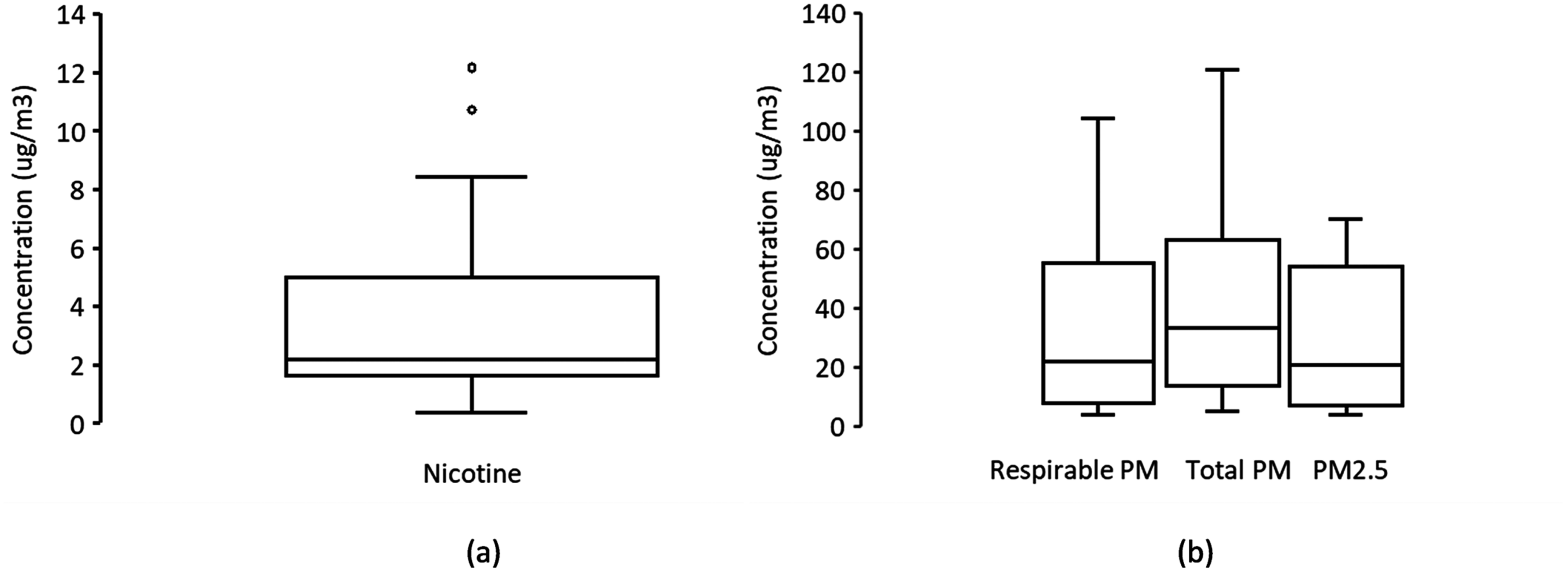
Boxplots for (a) nicotine and (b) PM_2.5_, respirable PM, and total PM across vape shops.

The average sampling period was 9.27 h and ranged from 7.00 to 12.00 h. The nicotine, PM_2.5_, respirable PM, total PM concentrations, and proportion of respirable PM within the vape shops are presented in Supplementary Table S5. The median and interquartile range are displayed in Fig. 1. Nicotine, PM_2.5_, and respirable PM data were not normally distributed, however, total PM was normally distributed. The geometric mean (GM) ± geometric standard deviation (GSD) for nicotine were 2.60 ± 2.62 ug/m^3^. Nicotine concentrations ranged from non-detects to 12.17 ug/m^3^. Nicotine was detected in 14 out of the 15 vape shops assessed. The field blank samples for nicotine were below detection limit.

The PM_2.5_ concentration ranged from 3.85 to 70.38 ug/m^3^ and had a GM ± GSD of 19.89 ± 3.06 ug/m^3^. The respirable PM had a range of 4.06 to 104.53 ug/m^3^ and GM ± GSD of 22.66 ± 2.95 ug/m^3^. The total PM had a mean ± SD concentration of 43.67 ± 34.78 ug/m^3^ with concentrations ranging from 5.11 to 120.98 ug/m^3^. Overall, respirable PM made up 76% of total PM in the measured vape shops. The 95th percentile for nicotine concentration (11.17 ug/m^3^) was 45-fold lower than the OSHA 8-h time-weighted average (TWA) permissible exposure limit (PEL) and ACGIH 8-h threshold limit value for nicotine (500 µg/m^3^)(ACGIH 2020). The 95th percentile values for respirable PM (82.82 µg/m^3^) and total PM (102.30 µg/m^3^) concentrations were also 60-fold and 147-fold lower than the OSHA PEL for respirable (5,000 µg/m^3^) and total (15,000 µg/m^3^) particulates, respectively. However, the 98th percentile value for PM_2.5_ levels (68.52 µg/m^3^) exceeded the US EPA 24-h PM_2.5_ standard of 35 µg/m^3^ by 2-fold.

Correlations between nicotine and the particle size fractions were not statistically significant; PM_2.5_ (*R*^2^ = −0.279; *P* = 0.314), respirable PM (*R*^2^ = −0.289; *P* = 0.295), and total PM (*R*^2^ = −0.307; *P* = 0.265). AER was not statistically associated with nicotine or total PM concentrations; nicotine (*R*^2^ = 0.078; *P* = 0.184) or total PM (*R*^2^ = 0.003; *P* = 0.330) (Supplementary Figs. S4 and S5). None of the observed variables in the shops were associated with nicotine concentrations (*P* > 0.05). Four variables significantly predicted total PM levels for a combined *R*^2^ of 0.4942, *P* = 0.0258. The significant predictors were: vaping in the shops, active ventilation, poor hygienic condition, and presence of activities that negatively impact indoor air quality. Vaping in the vape shops had a positive relationship with total PM levels while active ventilation, poor hygienic condition, and presence of activities that negatively impact indoor air quality had negative relationships with total PM levels (Supplementary Table S6). Poor hygiene conditions and activities that negatively impact indoor air quality were both expected to have a positive correlation with PM levels.

## Discussion

On average, VSWs in this study had vaped longer (6.75 ± 5.35 years) than in a study of EC users in France, Switzerland, and Belgium (4 ± 1 years) (Etter 2019). Multiple ECs were also maintained among VSWs that vaped (0 to 20, mean ± SD = 3.5 ± 5.3). One VSW did have a bucket of 20 ECs they selected from. Furthermore, number of puffs taken at work in this study ranged from 0 to 1,000 and averaged 398 ± 223 puffs which was higher than 44 to 345 (mean ± SD = 156.2 ± 10.3) reported in a study of EC users in Poland over a 24-h period (Kosmider et al. 2018). Compared to the Polish study which collected EC users puff data using a CReSS device, VSWs in this study self-reported number of puffs based on one of 3 approaches. (i) Their device kept track of the number of puffs, and they reported that to us. (ii) They estimated how many days it took for them to consume their disposable device that was supposed to have a known number of puffs. (iii) They did their best to estimate the number of puffs taken daily. E-liquid nicotine concentration reported by VSWs in this study ranged from 3 to 50 mg/mL compared to 1 to 24 mg/mL in Kosmider et al.’s (2018) study.

Most VSWs were former smokers and this was similarly reported in Joss et al.’s (2021) study. This was also in agreement with other studies on EC users (Etter 2010; Etter and Bullen 2011, 2014; Dawkins et al. 2013; Hart et al. 2016; Pattinson et al. 2018; Leavens et al. 2019; Galimov et al. 2021). A reason for this is that many tobacco users believe that ECs are healthier substitutes to traditional tobacco products (Richtel 2014) and therefore adopt ECs for their nicotine needs or as tools for quitting nicotine addiction or dependence altogether (Patel et al. 2016).

The majority of the VSWs engaged in workplace practices that are expected to negatively impact indoor air quality; such as vaping and permitting vaping by customers. Understandably, many of these VSWs were EC users prior to working as VSWs and they also use ECs to advertise to customers. Some customers may request to sample EC devices and products in-store before making purchases. Although some VSWs admitted that vape use was the most common air pollution source in their workplaces, most did not believe that there were activities impairing indoor air quality in their workplace including vaping and neither did their workplace environment adversely affect their health and well-being. Rather they rated the indoor air quality as near excellent. The objective assessment of PM and nicotine affirms that air quality within vape shops was quite good and well below any occupational exposure limits. However, the hierarchy of controls places elimination above engineering and administrative controls. In this situation, it is a better control to eliminate the primary identifiable source of indoor air pollution rather than depend on VSW judgment and general dilution ventilation.

The majority of the VSWs in this study agreed that elevated nicotine and PM levels, nicotine handling, and direct contact with nicotine and e-liquid were hazardous. However, most of the VSWs did not identify any hazards nor factors responsible for hazards in their workplaces. However, among those that reported workplace hazards; electrical hazards, slips, trips, and falls; and chemical handling were mainly identified. The presence of hazard controls in the workplace was not commonly reported by VSWs in this study and in vape shops where hazard control and prevention methods were present; they were reportedly initiated by the VSWs and their colleagues. Similarly, in (Garcia et al. 2016) most of the VSWs had experienced spillage of nicotine-containing e-liquid and had no training on nicotine handling. However, PPE (gloves and goggles) were reportedly provided for the majority of VSWs in Garcia et al.’s (2016) study, compared to half of VSWs in Attfield et al.’s (2022) study and only a quarter of VSWs in this study.

Onsite observations were performed to qualitatively evaluate indoor conditions at all vape shops in this study. All the vape shops in this study had no visible signs of haziness and only a few had odors even though vaping was commonly observed in most of the vape shops. This was in contrast to hazy indoor conditions reported in other studies by Attfield et al. (2022) in San Francisco, Son et al. (2020) in New Jersey and previously observed by this research group. Vaping was mainly performed by VSWs, and customer presence was low during the onsite observation period. Some vape shops also had “drive-through” options which were well utilized by customers. These could be responsible for the absence of haziness in the vape shops visited in this study.

More than half of the vape shops in this study had either partially or fully enclosed lounge areas where customers could sit, relax, and use ECs unlike vape shops in the Attfield et al. (2022) study with no separate vaping areas for customers. However, no customers were sighted in the lounge areas during visits. Workers in the vape shops were also observed not to use PPE and only a few vape shops had first aid kits and fire extinguishers; first aid kits and fire extinguishers are required by law. This was in agreement with other studies where the majority of vape shop workers did not use safety equipment (Garcia et al. 2016; Attfield et al. 2022). General ventilation was also present in all the studied vape shops, although a few vape shops did not use them at the time of observation which was similar to other studies (Nguyen et al. 2019; Son et al. 2020; Attfield et al. 2022). However, a study by Li et al. (2021a) in Southern California reported an absence of central ventilation in all 6 vape shops visited.

There was almost an equal proportion of large- (>250 m^3^) and small-sized (≤250 m^3^) vape shops in this study, in contrast to those studied Nguyen et al.’s (2019) and Attfield et al.’s (2022), which were mostly small-sized. Many shops had high air flow rates at their supply-air vents which is responsible for the higher AER observed in this study (0.1 to 9.7 h^−1^) compared to other studies such as Son et al. (2020) (0.126 to 0.152 h^−1^) and Li et al. (2021a) (0.21 to 1.06 h^−1^), which may be associated with the shop size.

The mean ± SD nicotine concentrations (3.92 ± 3.73 ug/m^3^) observed in this study were higher than those reported by Li et al. (2021a) (2.59 ± 1.02 ug/m^3^) over 24 h and Attfield et al. (2022) (1.73 ug/m^3^) over 8 to 12 h but well below the OELs. Mean nicotine concentrations in this study and the other similar studies were well below the OSHA OEL. Interestingly, mean ± SD PM_2.5_, respirable PM, and total PM (32.01 ± 25.85, 36.03 ± 30.91, and 43.67 ± 34.78 ug/m^3^, respectively) in this study were lower than PM_2.5_ levels reported by Son et al. (2020) and Li et al. (2021a) (1,660 ± 1,840 and 276 ± 546 ug/m^3^, respectively, at 24-h TWA). The 95th percentile values for PM in this study were also below their respective OELs. The 98th percentile for PM_2.5_ levels exceeded the EPA NAAQS for ambient PM_2.5_. Although the exposure levels observed in this study were well below OELs, it is important to remember that occupational exposure limits are intended for 8-h workdays within 40-h work weeks. If employee work shifts are longer than 8 h, then the exposure limit should be reduced using an acceptable approach such as the Brief and Scala model (Brief and Scala 1986; Verma 2000).

A similar study of 14 vape shops in Oklahoma City performed by Floyd et al. (Floyd 2017) in 2014, found much higher respirable PM and total PM levels (7,125 and 7,500 ± 1,500 ug/m^3^). This may be attributed to the shorter sampling period in that study (15 to 60 min) which could have coincided with periods of intense and more frequent EC use in vape shops at the time. In this study, 76% of total PM was respirable PM, compared to 95% in the Floyd et al. (Floyd 2017) study.

The low airborne nicotine and PM levels recorded in this study might be attributed to low customer traffic and reduced customer vaping in-store. Reduced customer traffic and vape use within vape shops might be due to the following factors; (i) The US Food and Drug Administration’s (FDA) “Tobacco 21” regulation (FDA 2019) restricts the sale of ECs to persons aged ≥ 21 years. During interviews, participants mentioned the negative effect of Tobacco 21 on the customer base, particularly among customers younger than 21 years. (ii) COVID-19 restrictions adversely impacted in-store customer visits, lounging, and social interactions. During the COVID-19 restrictions was the time that many vape shops embraced curbside deliveries and drive-through sales. (iii) The type of EC devices popularly used by the local vaping community has evolved to lower-powered, lower-emission devices such as disposables. Some of the factors mentioned were echoed by Duan et al. (2022) in a study assessing the impact of tobacco regulation and COVID-19 restrictions on vape shops in 6 US cities including Oklahoma City.

Furthermore, the negative correlations between nicotine and the PM size fractions in this study could imply that most of the PM originated from non-vaping activities in the shops, outdoor sources from opening and closing of the shops’ doors during customer visits and possibly cross-contamination from adjacent shops. Air exchange rate (AER) did not affect nicotine and total PM levels likely because nicotine and PM levels were too low to observe significant effects. The 4 significant predictors of PM levels were difficult to interpret. Both variables, poor hygienic condition and presence of activities that negatively impact indoor air quality were found to negatively correlate with total PM. This is counterintuitive since one naturally associates poor hygiene and recognized sources of air pollution with poorer indoor air quality. The other 2 variables were correlated as expected. Vaping in the shops was positively correlated with PM levels and active ventilation was negatively correlated with PM levels. These outcomes may indicate that having a well-functioning ventilation system is more important than cleanliness or identified activities that negatively impact air quality because there was no evidence that those identified activities were present during our measurements. However, due to sample size limitations, and rather low correlation (*R*^2^ = 0.494) the predictive value of these 4 significant factors is limited and warrants further investigation.

Our study is limited to assessing the building’s characteristics, VSWs’ perception of health risks, and work-hour exposure to nicotine and PM levels. Further studies on the vape shop’s aerosol’s chemical and toxic profiles and its biological impact elucidation will provide significant insight into WSV’s occupational-related health risk.

## Conclusion

The vape shop environments in this study did not pose a significant risk of second-hand exposure to elevated levels of airborne nicotine and PM during business hours. However, health and safety risks among VSWs are likely to arise from their EC use, perception of low workplace hazards, lack of health and safety training, and poor PPE use.

## Supplementary material

Supplementary material is available at *Annals of Work Exposures and Health* online.

wxaf018_suppl_Supplementary_Materials

## Data Availability

The data collected during this study is presented in their entirety in Supplementary Tables S1 to S6 and Supplementary Figs. S1 to S5.
