# Supplementary material for: Assessment of vape shop built environment: airborne nicotine, particulate matter, ventilation, hazard identification, workplace practices, and safety perceptions
Source: Ann Work Expo Health. 2025 Jun 19;69(5):510–9. doi: 10.1093/annweh/wxaf018 (PMC12208366; doi:10.1093/annweh/wxaf018)
Supplement: wxaf018_suppl_Supplementary_Materials [file wxaf018_suppl_supplementary_materials.pdf]

ASSESSMENT OF VAPE SHOP BUILT ENVIRONMENT: AIRBORNE NICOTINE, PARTICULATE MATTER, VENTILATION, HAZARD IDENTIFICATION, WORKPLACE PRACTICES, AND SAFETY PERCEPTIONS

Toluwanimi M. Oni MPH, Balaji Sadhasivam PhD, Evan L. Floyd PhD

Department of Occupational and Environmental Health, Hudson College of Public Health, University of Oklahoma Health Sciences, Oklahoma City, OK 73104, USA

Corresponding Author:

Evan L. Floyd PhD

Department of Occupational and Environmental Health,

Hudson College of Public Health,

University of Oklahoma Health Sciences,

Oklahoma City, OK 73104, USA

Evan-Floyd@ouhsc.edu

Table S1. Sociodemographic Characteristics of Vape Shop Workers

**Sociodemographic Characteristics**		**Proportion (%)**
Gender	Female	13
	Male	88
Race/Ethnicity	Middle Eastern	6
	Multiracial	6
	Native American	6
	White	81
Education	High School	50
	Some College	31
	Trade School	6
	Undergraduate	12
Annual Income ($)	10,000 – 19,999	25
	20,000 – 29,999	25
	30,000 – 39,999	12
	40,000 – 49,999	12
	50,000 and above	19
	Prefer not to answer	6

Table S2. Occupational History of Vape Shop Workers

Occupational History	Mean	Median	SD	Range
How long have you worked in a Vape Shop (years)?	4.57	4.00	3.09	0.3 - 10
How long have you worked in this Vape Shop (years)?	3.71	3.00	3.11	0.3 - 10
How long is your typical daily work shift (hours)?	8.19	8.00	2.40	4 - 15
How many hours do you work in a Vape Shop per week (hours)?	45.8	41.5	17.8	22 - 105

Table S3. Electronic Cigarette User Behavior among Vape Shop Workers

**Electronic Cigarette User Behavior**	**Mean**	**Median**	**SD**	**Range**
How long have you vaped (years)?	6.75	6.00	5.35	0 - 22
How many devices do you currently use?	3.5	2.0	5.3	0 - 20
How many times do you puff while working?*	398	350	223	100 - 1000
What is the setting of your most used device (watt)?	37.8	22.5	29.8	2.5 - 90
What is the concentration of nicotine you are vaping now (mg/mL)?	30.7	50.0	23.1	3 - 50

* Respondents found it difficult to answer this question accurately and reported a daily estimate for number of puffs based on one of three approaches. 1) their device kept track of the number of puffs and they reported that to us. 2) they estimated how many days it took for them to consume their disposable device that was supposed to have a known number of puffs. Ex: their device is rated for 5000 puffs and they replace it every week = 5000 puffs / 7 days = 714 puffs / dy. 3) They did their best to estimate how many puffs they take.

Table S4. Ventilation Parameters across Vape Shops

Vape Shop	Shop Volume (m^3)	Air Flowrate (m^3/min)	Air Exchange Rate (h^{-1})
1	520	31.9	3.7
2	227	36.5	9.7
3	394	NA*	NA*
4	163	16.6	6.1
5	1590	178	6.7
6	518	26.7	3.1
7	157	22.5	8.6
8	102	10.3	6.1
9	261	38.1	8.8
10	249	36.9	8.9
11	165	NA*	NA*
12	181	10.1	3.3
13	329	26.3	4.8
14	297	0.40	0.1
15	98	8.95	5.5

*Supply-air vents were not on at the time of measurement.

Table S5. Nicotine, $PM_{2.5}$, Respirable PM, Total PM and Respirable fraction concentrations across Vape Shops

**Location**	**Nicotine ($\mu g/m^3$)**	**$PM_{2.5}$ ($\mu g/m^3$)**	**RPM ($\mu g/m^3$)**	**TPM ($\mu g/m^3$)**	**PNC (particles/cm^3)**	**Respirable Fraction (%)**
1	1.99	53.32	55.59	69.61	16199.70	80
2	0.97	50.99	50.95	54.22	16006.92	94
3	8.45	59.36	59.59	64.28	18227.71	93
4	6.32	6.85	7.45	11.12	2266.03	67
5	10.74	5.81	6.85	12.16	1544.19	56
6	2.18	3.85	4.06	5.11	1077.47	79
7	1.52	20.75	21.88	25.40	6120.09	86
8	2.31	8.57	9.04	15.21	2531.57	59
9	3.18	55.17	55.23	59.89	17135.76	92
10	2.06	63.73	104.53	120.98	5052.08	86
11	1.04	70.38	73.51	94.30	17288.46	78
12	1.75	51.99	54.50	61.89	9794.80	88
13	ND (0.37)	6.94	8.51	15.27	2204.34	56
14	12.17	18.18	21.36	33.42	6301.57	64
15	3.71	4.24	7.33	12.13	314.63	60
Median	2.18	20.75	21.88			
GM	2.60	19.89	22.66			
GSD	2.62	3.06	2.95			

ND = Non-detect   GM = Geometric Mean   GSD = Geometric Standard Deviation

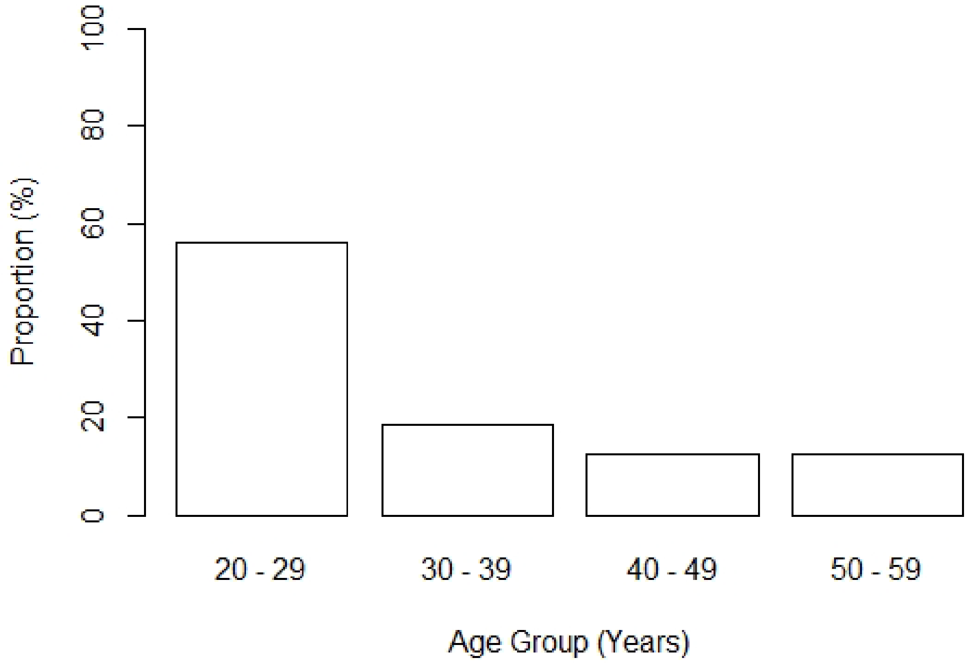

Figure S1. Age Groups of Vape Shop Workers

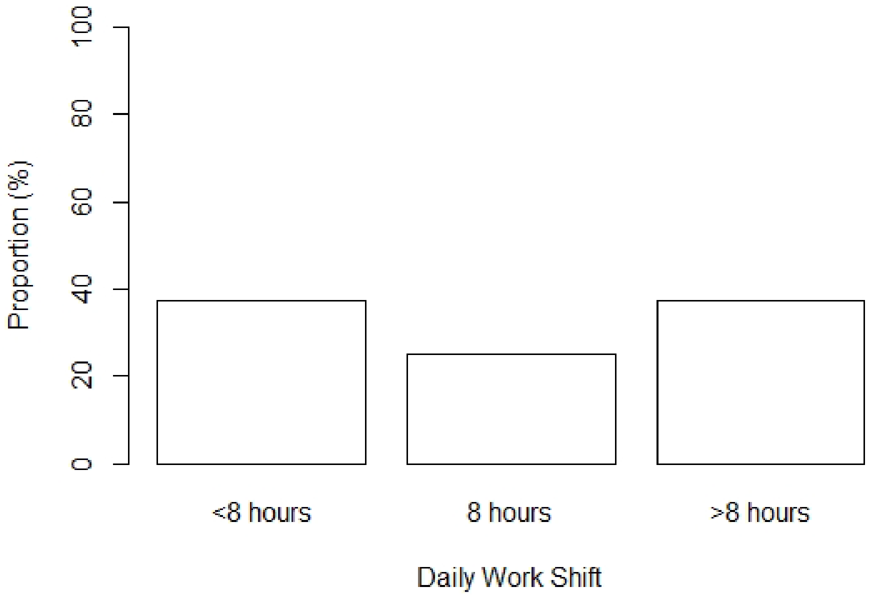

Figure S2. Daily work shift of Vape Shop Workers

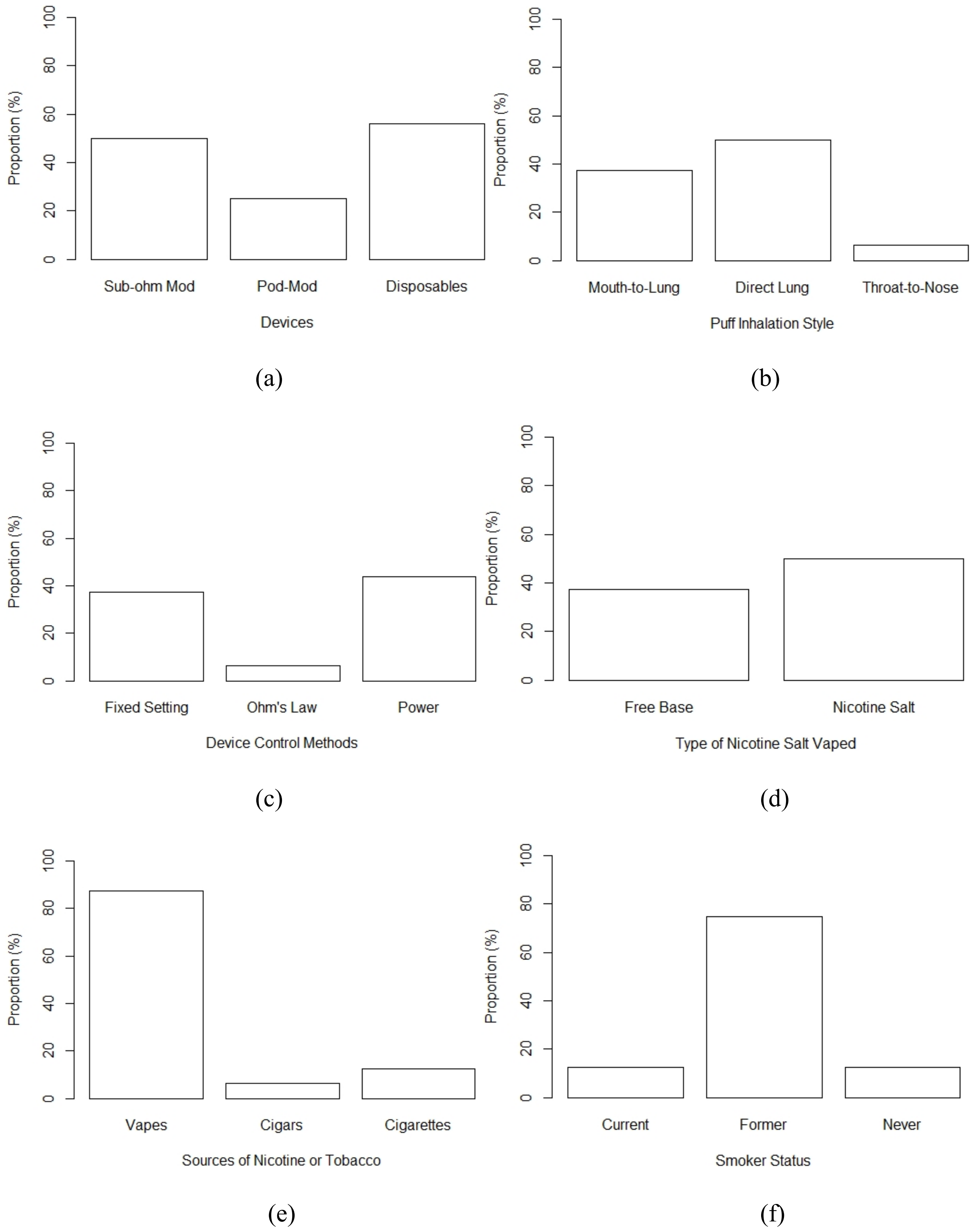

Figure S3. Electronic Cigarette Use Behavior among Vape Shop Workers (a) Types of Electronic Cigarette Devices Commonly Used (b) Puff Inhalation Styles (c) Device Control Techniques (d) Type of Nicotine Vaped (e) Sources of Nicotine or Tobacco Regularly Used (f) Smoker Status

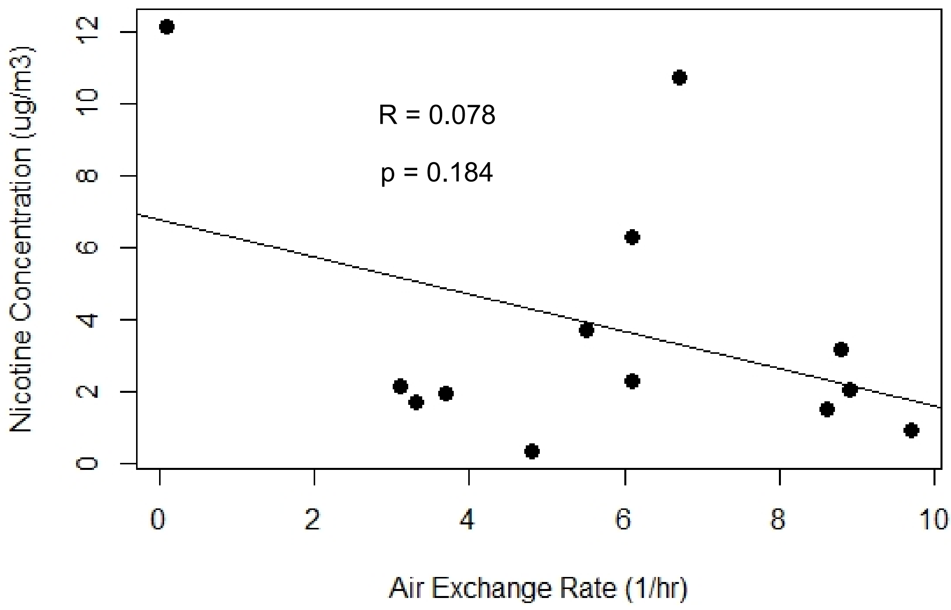

Figure S4. Linear Regression plot of Nicotine Concentration and Air Exchange Rate (AER)

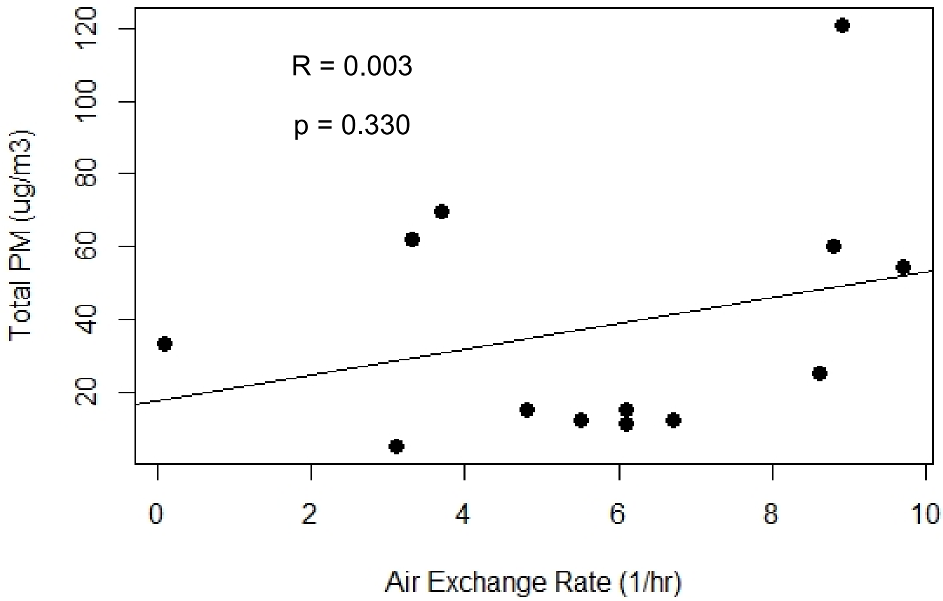

Figure S5. Linear Regression plot of Total PM Levels and Air Exchange Rate (AER)

Table S6. Multivariate Regression Analysis Showing Significant Predictors of Total PM levels in Vape Shops

	Estimate	Error	t-value	p-value	Adjusted R^2	Overall p-value
Intercept	78.77	18.89	4.169	0.00192	0.4942	0.0258
Active Ventilation	-50.23	19.16	-2.622	0.02553		
Poor hygienic condition	-80.09	30.29	-2.644	0.02458		
Presence of activities that negatively impact indoor air quality	-65.71	20.45	-3.214	0.00927		
Vaping in the shops	66.75	20.45	3.265	0.00851		

OBSERVATIONAL CHECKLIST

Location ______________ **Time** ________________ **Date** ______________________

Air Quality

S/N	Characteristics	YES / NO
1	Is there visible PM or haze?	
2	Presence of odors or flavors?	

3. List sources of indoor air pollution within the vape shop

____

____

____

Building Characteristics

1. Length ______________ 2. Width _______________ 3. Height ______________

4. Ventilation type (General/Local) _________________ 5. If local ventilation, how many? _____

6. Number of air vents _____________ 7. Air flow rate from vents _______________

8. Number of air filtration systems ______________ 9. Number of ceiling fans _______________

10. Additional observations of ventilation systems

____ _____

S/N	Characteristics	YES / NO
6	Is there a lounge area in the shop?	
7	Is the lounge area partially or fully enclosed from the shop area?	

10. Rate the hygienic condition of the vape shop. (Good, Fair and Poor). ____

Presence and Use of Safety Equipment

S/N	Equipment	Absent	Present	Number if Present
1	Face Mask			
2	Aprons			
3	Gloves			
4	Goggles			
5	Safety signs			
6	First Aid kits			
7	Other			
